# Ending the HIV Epidemic: Identifying Barriers and Facilitators to Implement Molecular HIV Surveillance to Develop Real-Time Cluster Detection and Response Interventions for Local Communities

**DOI:** 10.3390/ijerph20043269

**Published:** 2023-02-13

**Authors:** Moctezuma Garcia, Samantha Devlin, Jared Kerman, Kayo Fujimoto, Lisa R. Hirschhorn, Gregory Phillips II, John Schneider, Moira C. McNulty

**Affiliations:** 1 Department of Social Work, College of Health & Sciences, San José State University, San Jose, CA 95112, USA; 2 The Chicago Center for HIV Elimination, University of Chicago, Chicago, IL 60637, USA; 3 Department of Health Promotion & Behavioral Sciences, University of Texas Health Sciences Center, Houston, TX 77030, USA; 4 Department of Medical Social Sciences, Feinberg School of Medicine, Northwestern University, Chicago, IL 60611, USA; 5 Department of Medicine, University of Chicago, Chicago, IL 60637, USA

**Keywords:** Ending the HIV Epidemic, cluster detection and response, molecular HIV surveillance, public health, molecular epidemiology, qualitative research

## Abstract

The rapid implementation of molecular HIV surveillance (MHS) has resulted in significant challenges for local health departments to develop real-time cluster detection and response (CDR) interventions for priority populations impacted by HIV. This study is among the first to explore professionals’ strategies to implement MHS and develop CDR interventions in real-world public health settings. Methods: Semi-structured qualitative interviews were completed by 21 public health stakeholders in the United States’ southern and midwestern regions throughout 2020–2022 to identify themes related to the implementation and development of MHS and CDR. Results for the thematic analysis revealed (1) strengths and limitations in utilizing HIV surveillance data for real-time CDR; (2) limitations of MHS data due to medical provider and staff concerns related to CDR; (3) divergent perspectives on the effectiveness of partner services; (4) optimism, but reluctance about the social network strategy; and (5) enhanced partnerships with community stakeholders to address MHS-related concerns. Conclusions: Enhancing MHS and CDR efforts requires a centralized system for staff to access public health data from multiple databases to develop CDR interventions; designating staff dedicated to CDR interventions; and establishing equitable meaningful partnerships with local community stakeholders to address MHS concerns and develop culturally informed CDR interventions.

## 1. Introduction

In 2018, the Centers for Disease Control and Prevention (CDC) required health departments that received HIV funding to integrate molecular HIV surveillance (MHS) for HIV cluster detection and response (CDR) among priority populations [1,2]. An HIV molecular cluster is composed of people living with HIV (PLWH) that have a highly similar HIV genetic sequence [1,3,4]. CDR requires health departments to identify, assess, and monitor molecular clusters in real time to prioritize tailored prevention responses based on cluster characteristics [5]. The United States government’s plan titled Ending the HIV Epidemic (EHE) has invested in MHS as a core component towards integrating CDR to enhance access to HIV-related services for people associated with a molecular cluster in highly burdened jurisdictions [1,6,7,8,9]. Health departments have rapidly integrated MHS as a tool for CDR, which has resulted in enhanced staff training and development [3].

In the United States, association with a molecular cluster has raised concerns related to HIV criminalization exposure laws that are still reinforced in some states if a PLWH exposes another person to HIV [10,11]. Significant challenges related to the implementation of MHS and development of CDR have been due to community concerns regarding government mistrust, informed consent, stigmatization, and HIV criminalization [11,12,13]. Despite the rapid development of MHS [14], limited research is available on the implementation of MHS to develop real-time CDR interventions at local health departments in the United States [15]. Public health professionals have expressed a need for refined training on MHS to address the needs of people associated with molecular clusters [16]. HIV genetic sequences are also not always available for PLWH [16], which results in missing data for molecular clusters. Health departments must take into account implications related to the transparency of MHS data collection and meaningful community engagement among impacted populations [17]. An emphasis was placed on the need to train Disease Intervention Specialists (DIS) to engage people associated with molecular clusters and address MHS-related issues (e.g., HIV criminalization, medical mistrust) [17]. To date, several key questions remain around the implementation of MHS to improve partner services [18,19,20] for identifying (1) people newly diagnosed with HIV; (2) PLWH with a detectable viral load; and (3) people vulnerable to HIV exposure. A qualitative study was administered to ask stakeholders involved in MHS-related activities the following question: How do MHS-related barriers and facilitators enhance initiatives for CDR?

## 2. Materials and Methods

An exploratory, qualitative research design was developed to explore barriers and facilitators associated with the implementation of MHS to develop real-time CDR interventions for local communities. Professionals involved in MHS-related activities with health departments in the South and Midwest regions of the United States completed in-depth qualitative individual interviews from February 2020 through February 2022. The Institutional Review Board at the University of Chicago approved the study and participants verbally consented to be involved in the study.

### 2.1. Sampling

A planning committee was established for this study and purposive sampling was used to identify professionals collaborating with health departments addressing HIV. The planning committee identified professionals (e.g., public health, community-based organizations, government) collaborating with local public health departments addressing HIV in the South and Midwest. Potential participants were contacted by email and/or phone to participate in the study. Eligibility criteria for this study required participants to have some knowledge of MHS- or CDR-related activities.

### 2.2. Interview Guide and Data Collection

An interview guide (Supplement S1) was developed to explore the following domains on how MHS-related activities enhance initiatives for CDR: (1) implementation of MHS-related activities; (2) MHS data utilization for CDR; (3) social network strategy (SNS) to supplement CDR; (4) partner services to supplement CDR; and (5) community engagement of stakeholders to enhance CDR. Only two interviews (45–60 min) were conducted in person and audio-recorded; due to COVID-19, many of the interviews were completed through teleconferencing. All participants were offered a USD 100 gift card for completing the study. Interviews were completed once saturation was reached and no new information was obtained related to the social phenomenon of interest [21].

### 2.3. Data Analysis

A thematic analysis was done based on Braun and Clark’s [22] following phases: (1) becoming familiar with the data; (2) generating codes; (3) searching for themes; (4) reviewing themes; (5) defining themes; and (6) producing the report. Interviews were digitally recorded and transcribed verbatim. NVivo [23] software was used to complete the thematic data analysis. The research team became familiar with the data by reviewing, cleaning, and removing any identifiable information. The research team met on a regular basis to review the initial codes, establish a refined codebook, and discuss initial themes. Once the themes were clearly defined, a report was drafted to prepare the results for the thematic analysis. Unique identifiers were assigned to each participant and are used for direct quotes throughout the results section to protect the identity of participants. Any additional identifiable information (e.g., names) disclosed throughout the interviews was removed from the transcripts to ensure confidentiality. 

## 3. Results

A total of 21 participants in the United States (12 in the South and 9 in the Midwest) completed this study; refer to Table 1 for participant demographics. 

### 3.1. Themes

Five prominent themes emerged: (1) strengths and limitations of using HIV surveillance data for real-time CDR; (2) limitations of MHS data due to medical provider and staff concerns related to CDR; (3) divergent perspectives on the effectiveness of partner services; (4) optimism, but reluctance about the social network strategy (SNS); and (5) enhanced partnerships with community stakeholders to address MHS-related concerns. 

#### 3.1.1. Strengths and Limitations in Utilizing HIV Surveillance Data for Real-Time CDR

All participants provided an overview on how HIV surveillance is an integral part of CDR in utilizing data to identify and prioritize molecular clusters for investigation. Participant 17-South indicated that, during cluster detection, “…if we see that there are an exceptional amount-number of cases… we would implement a rapid response” and “…look at the data that’s coming from our surveillance unit”. Participant 12-Midwest elaborated on the importance of HIV surveillance data for CDR and collaborating with colleagues to better understand the characteristics of people associated with the molecular cluster:

*So, the data is analyzed by our HIV surveillance and epidemiology team. They run this data and whenever a new cluster is identified then that’s sent to my team to follow up with those individuals who break the cycle of transmission. When we identify a cluster, those individuals they’re sent to the HIV partner services team that we would assign for field investigations and we’ve worked with those clients to understand their partners and notify their partners in that cluster*.

However, participants indicated that CDR requires health departments to gather data from multiple databases to tailor the response based on characteristics of people associated with the molecular cluster. Health departments do not have a central database to easily access the characteristics of people associated with the molecular cluster, and staff struggle when using outdated systems to collect data to deepen their understanding of cluster characteristics, which hinders their progress to tailor real-time CDR interventions. Participant 12-Midwest indicated that a significant barrier is that they do not have a central database, which requires “…pulling data from those different sources and allowing us to run reports so that we can better understand what’s happening with clients”. Participant 14-South stated that “...we have like 8 or 10 different sources that have not been integrated. We first have to make sure that all the data downloaded from these [databases], each of them match. And some of them don’t have a common ID”. Participant 4-South shared their experience in retrieving client information from multiple databases:

*So, the biggest challenge is that different systems are housed in different bureaucratic areas, different systems that necessarily share unique identifying variables. So, for example, I don’t have actual access to eHARS and there is no variable in the counseling and testing system that matches a variable in eHARS. So, we have to do kind of a fuzzy match based on name and date of birth to try to connect testing data to surveillance data*.

#### 3.1.2. Limitations of MHS Data Due to Medical Provider and Staff Concerns Related to CDR

Health departments are dependent on medical providers to collect and submit HIV genetic sequences for CDR to be successful in identifying molecular clusters. However, medical provider standards of care may not require HIV drug resistance tests to treat newly diagnosed PLWH, or there may be delays in submitting HIV genetic sequences due to unforeseen events (e.g., COVID-19), as shared by participants from both regions:

*The CDC has requirements for the number of genetic sequences that you’re supposed to be receiving in your area. Towards the end of last year, we noticed that there was a decrease. And this was even before COVID. I think a large part has to do with providers not ordering as many genetic sequences. Because in the past, they would use that information to kind of tailor their treatment protocols. But now with the treatments for HIV being improved, they don’t need that information from the genetic sequences. *

(Participant 16-South)

*I mean it’s probably been a consistent message that just timeliness of data is getting providers to report. Labs have automated reporting for the most part, that’s pretty quick and timely and that’s really improved in the past few years and made a world of difference. And then you put COVID on top of all of that and it’s a challenge.*

(Participant 18-Midwest)

Despite health department staff acknowledging the significance of MHS, many participants in the South questioned the effectiveness of CDR. They recommended further pilot testing to compare the efficacy of CDR. Participant 15-South emphasized, “I think we’d have to analyze it [CDR] and see how successful was it in our traditional partner services program and then make the decision”. Participant 4-South questioned whether CDR is valuable when comparing it to partner services: “I think in a jurisdiction where there is already universal surveillance-based partner services is when it’s [CDR] useless”. However, Participant 17-South indicated, “I think the science is there…” for identifying clusters and saw the value, but acknowledged “…we had to go back and develop the intervention around it, which wasn’t that difficult, because we’ve already had done some things in the past”.

#### 3.1.3. Divergent Perspectives on the Effectiveness of Partner Services

Once a molecular cluster has been identified and prioritized, partner services data provide a critical source of information to expand the molecular cluster and include partners that may benefit from HIV-related services. Participant 13-South indicated, “Partner Services is… a proven intervention that reduces the spread of HIV and other sexually transmitted diseases”. The following participants emphasized the importance of integrating partner services with CDR.

*We use the* [partner service] *data to identify the molecular clusters and then, the underlying transmission clusters, so, then we can develop high impact interventions or prevention services. We can get individuals linked or re-linked into care. Offer... people that test negative, connected with PrEP services. The goal is to stop the disease transmission.*

(Participant 16-South)

*…I think there is* [epidemiological] *data that partner services is uniquely able to collect. I talked about that at the beginning: risk information, substance use, psychosocial. They also can connect because partner services in almost every jurisdiction does try to touch or connect with every person who has got an HIV diagnosis or a new syphilis diagnosis and other jurisdictions might do other things like a gonorrhea, etcetera.*

(Participant 18-Midwest)

All health departments indicated that partner services have enhanced CDR approaches for increasing access and engagement to HIV services for underserved populations. However, there were significant differences between health departments on limitations associated with partner services. Health departments in the Midwest were more likely to emphasize limitations due to the quality of data, eliciting partners, and timeliness of partner services data. Participant 2-Midwest emphasized “…that [Partner Services] data is probably the worst quality data that the [Health Department] has.” Participant 12-Midwest stated, “We’ve had not great success in eliciting partners with people” through partner services because “…many people are meeting a lot more anonymous partners and do not have the information for their partners”. The timeliness of partner services data was another barrier related to CDR, raised by Participant 19-Midwest, that has hindered staff’s capacity to engage newly diagnosed PLWH into services:

*…it’s more often than not, the HIV cases that are generated for partner services interview are too old. There have been times when a nine-month-old case is assigned to a disease intervention specialist to conduct an interview. And this is a person that was diagnosed with HIV nine months ago has done whatever he/she or they did after learning status hopefully got into care, has made peace, and is living life and then the government calls saying, “We want to talk to you and we want you to give us your sexual partners’ names”. It kills our credibility and doesn’t provide us really good data. So, the timeliness between an actual positive test result and the partner services interview is really critical.*

(Participant 19-Midwest)

#### 3.1.4. Optimism But Reluctance about SNS

All health departments viewed SNS as an important opportunity to overcome barriers related to partner services. Participant 12-Midwest stated, “I guess social network strategy is not given much attention and I would love to expand that. But molecular clustering and partner services are definitely high priorities”. The following participant stated that a primary barrier is not having knowledgeable staff dedicated to the implementation and development of SNS for CDR. Participant 20-Midwest: “So, it’s not part of the routine for us… and again, another thing to consider is that we do not have a person who has lots of experience doing social network analysis in-house”. The following quote from Participant 15-South indicates how SNS provided access to HIV testing for people not accessed through traditional services:

*I think it’s a great tool* [SNS]*, and that I’m absolutely optimistic and want to continue to see if it brings in, or if we find new positives. I mean, we were happy to see that people who’ve never tested before. Our population, of course it was the Latino population. So, we assumed that there were a lot of undocumented people who maybe would have not had the opportunity to test or were afraid to go anywhere to test, so that was positive…I think that I’m hopeful and I’m waiting to find* [newly diagnosed PLWH].

(Participant 15-South)

However, some barriers associated with SNS were due to having staff training to support SNS recruiters, as specified by Participant 14-South: “Like the amount of time you have to spend with each of these recruiters, the amount of follow-up… this is an added time for them [DIS]”. Health departments in the South indicated that the implementation of SNS for CDR placed a greater demand on staff. Participant 4-South stated, “it’s [SNS] a whole lot of work to essentially just find an average person that we’re gonna ask, who do you think we should test? We already do that without putting in all that front-end work, through traditional partner services”. Participant 14-South shared their thoughts about the complexity of supplementing CDR with SNS:

*So, there’s quite a bit of upfront commitment for SNS. I’m guessing…a cluster looks different each time. Your planning has to change every* [time]*, you know, if it looks drastically different, like I can’t do the same things or say the same things if it’s a predominantly Hispanic, Latino, you know cluster versus a diverse younger cluster, right? So, there’s quite a bit of upfront commitment. So, you do need staff who are already trained on it that you could pull from.*

Health department staff in the South indicated that SNS has increased HIV testing for people associated with the molecular cluster, but they were unable to find people that were undiagnosed. They wondered if the amount of work to implement SNS is an effective approach to strengthen CDR in mitigating HIV transmission outbreaks. Participant 14-South stated, “I was really hopeful that we will be able to get new diagnoses with SNS, but that hasn’t happened. Now we did test a lot of people”. Participant 8-South expressed that “it doesn’t have to be the method” for CDR because they were unable to find newly diagnosed PLWH associated with the molecular cluster.

#### 3.1.5. Enhancing Partnerships with Community Stakeholders to Address MHS-Related Concerns

Health departments emphasized the importance of community engagement and enhancing trust by establishing partnerships with local community stakeholders for a successful CDR program. Participant 4-South stated, “We were very involved at every single step of the way in ensuring that we were transparent with the community, having meetings, getting input”. Participant 16-South emphasized, “Make sure that they [community] feel included in the decision-making, that they’re included in the whole part of the project from the start through the end. So, that way they don’t feel marginalized”. As the participants indicated, an emphasis has been placed by health departments on providing a space to inform and engage with the community to identify best approaches on how to proceed with CDR. Participant 12-Midwest further indicated,

*I think that if we were able to take on a more peer-to-peer centered approach, I think that would help in people being more receptive to speaking with the health department if they see themselves and the people who are reaching out to them. Building that out would be important. I think just having the resources and capacity to offer real services within the field would help build trust and also acceptable at this type of program within the community and having more community members and providers just talk about the interventions and be clear about what the interventions are and how they can benefit their clients, their communities, their peers would be helpful and building that trust.*

Despite efforts by health departments to establish partnerships and enhance trust among the community, government mistrust raises concerns about being transparent on how HIV genetic sequences are being collected and the rationale for promoting MHS as a tool to enhance access to and engagement with HIV-related services. Participant 8-South elucidated on the complexity of administering CDR and addressing community concerns: “Our community is very up in arms about the privacy violations and potential criminalization of using this data. The huge worry by the community plus unknown effectiveness, it’s really hard to make a case for this kind of intervention continuing”. Participant 12-Midwest expressed that “…the biggest barrier is trust” and it’s important for the health department to be transparent “…about how data’s collected, how data’s used and the services that we’re able to provide to the client and their partners.” Participant-19 Midwest further elaborated:

*It’s* [CDR] *created a lot of concern for advocates and activists in many other jurisdictions… there are concerns about the abilities or the perception that cluster analysis can determine directionality of infection and people’s concern that they may be sort of identified as a person that infected someone else and are there legal implications and criminalization implications that are associated with data that might be able to tell that I was the person that infected that person. The data don’t do that. They don’t determine directionality, so that is just a perception. But that is the perception of what MHS could do and the danger that it could potentially cause is something that folks are worried about.*

At the time of this study, all participants indicated that the community had heightened concerns related to HIV criminalization for PLWH. The South also specified immigration issues for foreign-born populations. Participant 16-South stated, “… they’re probably less likely to engage in testing or care from a government agency because they don’t want to identify themselves and they’re afraid of being tracked and what it could mean for themselves and their families”. Participants expressed the need for conscious approaches by health departments that address community concerns and emphasize benefits related to CDR.

## 4. Discussion

The development of real-time response systems using MHS to support CDR is a core element of the EHE “Respond” pillar [24]. This study is among the first to explore public health professionals’ strategies to implement MHS and develop CDR interventions within real-world public health settings and methods for ongoing network-based strategies (e.g., partner services, SNS). The challenges and opportunities identified may inform health departments’ future development of CDR.

CDR is rooted in public health investigation [25], based on additional information about sexually transmitted infections and partner services data. Our research findings (Themes 1 and 2) from participants describe the difficulty of using HIV surveillance data from multiple databases due to incomplete reporting, poor data quality, the inaccessibility of data, and an inability to easily link surveillance data. Some of these administrative and bureaucratic barriers echo challenges and criticisms of the United States public health system exposed during the COVID-19 pandemic, including decentralization, a lack of public health infrastructure, and a disconnect from health systems, all of which will require the modernization of the system in order to be overcome [26,27]. Our participants highlighted many logistical challenges faced in coordinating real-time CDR, especially when integrating partner services (Theme 3) and SNS (Theme 4). Many of the HIV surveillance and prevention public health personnel were reassigned to the COVID-19 response and will be the same workforce that is tasked with responding to the next epidemic. Developing a functional and efficient response system in which surveillance and other data systems are integrated for use by an adaptable and pluripotent workforce will be crucial to control not only the HIV epidemic, but also future epidemics.

Since its inception, MHS has been fraught with objections and ethical concerns from community members due to a lack of transparency and consent to use phylogenetic data for CDR; its potential for use in establishing the directionality of transmission; and possible criminalization, the potential loss of privacy, and the perpetuation of stigma in already marginalized communities [17,28]. Researchers indicated that community and provider concerns remained despite taking a transparent and proactive approach involving the dissemination of MHS-related information to the Ryan White Planning Council, sharing information on its website, and alerting community members to clusters with multidrug-resistant HIV [17]. Our research findings for Theme 5 demonstrate that these concerns also impact public health professionals carrying out CDR, with this being a prominent concern expressed by our research participants. Community engagement around MHS-related activities has largely been left to individual health departments, but a centralized approach to disseminating MHS-related information and promoting equitable community engagement to all stakeholders may be beneficial, including those within the field of public health [17]. Approaches that center on meaningful community involvement will be crucial to alleviating these concerns and thus improving the success of CDR [17,29].

Community concerns were prominent in the South and the Midwest of the United States. In addition to the impact of the political environment across the regions, it is likely that the timing of the interviews played a role. As these interviews were being conducted, highly publicized immigration crackdowns and the weakening of sexual and gender minority protections were occurring simultaneously with the heightened distrust of American institutions exacerbated by COVID-19 misinformation and mixed public health messaging [30]. Further, differences in criminalization laws exist between the Midwest and Southern jurisdictions included in this study. Some of the regional differences may also be explained by variations in partner services delivery across regions. One of the Midwest public health jurisdictions included in this study funded community-based organizations (CBOs) to perform partner services, while the South performed all partner services directly within the health department. Partner services delivered by CBOs may engender trust and be better received by community members. However, sharing and receiving data with external partners creates additional complexity in responding to identified clusters, involving another layer of communication around prioritization and additional data sources to be incorporated into the process.

A recent systematic review has indicated that few interventions exist that delineate the best practices and effectiveness of CDR [15]; our participants suggested that protocols have been homegrown within local health departments. For CDR to be actionable, health departments need clear information and an understanding of how to respond to such information. Public health officials have noted that protocols must be in place for effective CDR interventions [16]. A more standardized approach will be crucial for effectively scaling out CDR and achieving a public health benefit for EHE. However, this may not be achievable until community concerns are sufficiently addressed, as some research has been paused or halted as a result [31]. Further, incorporating necessary services that address community needs into CDR interventions will be imperative for building trust.

Overcoming challenges in implementing MHS and real-time CDR requires program evaluation, evidence-based approaches, and close collaboration with community stakeholders. A perpetual challenge in implementing HIV phylogenetic monitoring is balancing the need to protect an individual’s right to privacy and the public health department’s responsibility to prevent HIV transmission [14]. The meaningful engagement of community stakeholders with the local health department fosters trust and enhances MHS-related activities addressing concerns in the community about privacy and confidentiality—including the fear of naming their sexual partners [32]. The administration of MHS demands continuous program evaluation and implementation science to inform best practices for identifying acceptable evidence-based approaches within specific contexts [33]. Despite potential challenges, many programs have produced results demonstrating that real-time sequencing is feasible and the obtained HIV phylogenetic data can help to identify previously unknown segments of transmission networks, overcoming the issue of unnamed partners [34]. Peer-based interventions such as SNS provide an opportunity for people in the community to play an active role in enhancing access and engagement to HIV-related services, but greater research is needed to determine if this is an effective approach to supplement CDR interventions.

There are several limitations to this study. First, the qualitative research findings are not generalizable due to the small sample size, but the in-depth interviews provide critical insights towards the development and implementation of MHS for real-time CDR interventions based on the knowledge and direct experience of professionals with partner services, SNS, MHS, and CDR. First-hand perspectives of community members were not included, and future research is needed to further enhance MHS-related activities that engage community stakeholders. Due to the COVID-19 pandemic, data collection for this study occurred over a longer period due to the availability and competing priorities of public health professionals. COVID-19 also interrupted CDR activities within health departments. 

## 5. Conclusions

Findings for this study emphasize that health departments require greater support to successfully accomplish the government’s plan to promote MHS as a tool to develop real-time CDR interventions for EHE among priority populations impacted by HIV. Implications for public health include developing an integrated centralized system of multiple existing databases within the health department to identify the characteristics of people associated with the molecular cluster to tailor real-time CDR interventions. The CDC should enhance its efforts to encourage medical providers to collect HIV genetic sequences, as well as augment multi-stakeholder (e.g., community, public health staff) buy-ins. Local health departments are encouraged to establish equitable, inclusive, and meaningful partnerships with community stakeholders to play an active role in developing culturally relevant interventions for EHE within their community. Lastly, evidence-based research findings should be disseminated among multi-stakeholders to demonstrate that MHS and CDR is more efficient and cost-effective when compared to existing interventions.

## Figures and Tables

**Table 1:** Participant demographics (*n* = 15 *).

Variable	*n* (%)
Region	
South	8 (53.3)
Midwest	7 (46.7)
Years of Experience	
0–4 years	6 (40.0)
≥5 years	9 (60.0)
Age	
18–29	2 (13.3)
30–39	5 (33.4)
40–49	3 (20.0)
50–59	3 (20.0)
≥60	2 (13.3)
Race/Ethnicity	
White, Non-Hispanic	11 (73.3)
Hispanic	3 (20.0)
Other	1 (6.7)
Gender	
Cisgender Female	7 (46.7)
Cisgender Male	7 (46.7)
Other	1 (6.7)

* 15/21 participants completed the demographic survey.

## Data Availability

The data that support the findings of this study are available on request from the corresponding author. The data are not publicly available due to privacy or ethical restrictions.

## Supplementary Materials

The following supporting information can be downloaded at: https://www.mdpi.com/article/10.3390/ijerph20043269/s1, Supplement S1. Semi-structured interview questions exploring how MHS-related activities enhance initiatives for CDR.
