# Supplementary material for: Ending the HIV Epidemic: Identifying Barriers and Facilitators to Implement Molecular HIV Surveillance to Develop Real-Time Cluster Detection and Response Interventions for Local Communities
Source: Int J Environ Res Public Health. 2023 Feb 13;20(4):3269. doi: 10.3390/ijerph20043269 (PMC9964218; doi:10.3390/ijerph20043269)
Supplement: Supplementary file 1 [file ijerph-20-03269-s001.zip › ijerph-2134998-supplementary.pdf]

Supplement S1. Semi-structured interview questions exploring how MHS related activities enhance initiatives for CDR.

I. Implementation of MHS Related Activities

1. Do you expect to have the sufficient resources (e.g., training, staff) to adopt, implement, and sustain additional tools for engaging people in molecular/phylogenetic clusters in your organization? *[probe to describe further facilitators and barriers related to MHS]*
2. What kind of local, state, or national policies will help or hinder the decision to adopt tools (e.g., software, data systems, consultation) for working with cluster outbreaks? [probe to describe further facilitators and barriers related to MHS related data]
3. How does the current system work for responding to new clusters of HIV?
4. If you had to develop a system to respond to new clusters of HIV, how would you respond?
5. What would this type of system need to function appropriately?

II. MHS data utilization for CDR

6. How would you use additional cluster outbreak data for your work?
7. Do you think additional cluster outbreak data could be used to meet the needs of identifying new individuals living with HIV?
8. What do you see as the advantages and disadvantages to using cluster data for identifying new individuals living with HIV?
9. Who within your organization would need to be involved to make decisions about implementing new approaches to using molecular cluster data?
    - *Probe: Do you think your organization would be receptive to implementing new approaches to using molecular cluster data?*

III. Social Network Strategy to Supplement CDR

10. From your perspective, describe how useful Social Network Strategy has been in addressing the HIV epidemic at your organization. *[refer to SNS language above to prompt]*
11. How confident do you feel that adopting additional strategies like the social network strategy will help in finding new people living with HIV or people who need PrEP?
12. What are some of the barriers to adopting the social network strategy or similar approaches?
    - *Probes to describe further barriers and facilitators related to SNS:*
        - What are some of the barriers to finding new people living with HIV using the social network strategy or similar approaches?
        - What are some of the facilitators to adopting the social network strategy or similar approaches?
        - What are some of the facilitators to finding new people living with HIV using the social network strategy or similar approaches?

IV. Partner Services to Supplement CDR

13. From your perspective, describe how useful partner services has been in addressing the HIV epidemic at your organization.

14. How do you think your organization would be most successful at using a new data source for partner services?

V. Community Engagement of Stakeholders to Enhance CDR

15. Describe current collaborations or partnerships between your organization and others around sharing of data, like partner services. What about sharing of molecular/phylogenetic cluster information?
16. Are there existing partnerships that have been helpful for successful implementation of partner services or social network strategy? *[probe - examples would be partnerships for data sharing or case referral]*
17. What have been some of the successes and challenges of the existing strategies?
18. Are there additional partnerships that would need to be established for successful implementation of partner services or social network strategy?
